# Childhood Sexual Abuse and Common Mental Health Problems: Do Sexual Trade and Exchange Mediate the Relationship?

**DOI:** 10.1007/s40653-026-00846-x

**Published:** 2026-03-11

**Authors:** Marianne Nilsen, Mari K. Gaardholm, Tormod Rimehaug

**Affiliations:** 1 Department of Social Work, Norwegian University of Science and Technology (NTNU), Trondheim, Norway; 2 Child Protective Services, Oslo, Norway; 3 Regional Centre for Child and Youth Mental Health and Child Welfare, Norwegian University of Science and Technology (NTNU), Trondheim, Norway

**Keywords:** Sexual trade and exchange, Anxiety, Depression, Substance abuse, Residential youth care

## Abstract

Childhood sexual abuse (CSA) is likely to have significant implications for mental health outcomes across the lifespan. This study examines the relationship between CSA, sexual trade and exchange (STE), and common mental health problems (MHPs) among 335 adolescents (aged 12–20) as part of a larger study on adolescents in Norwegian residential youth care (RYC). We investigated the mediating role of STE in the relationship between CSA and MHPs, such as anxiety, depression, and substance abuse, using the Child and Adolescent Psychiatric Assessment (CAPA). We found that STE mediated the relationship between CSA and substance abuse. CSA was directly associated with anxiety and depression, while STE was significantly associated with anxiety, but not with depression. This study highlights the significant role of STE in association with substance abuse among sexually abused adolescents and underscores the need for further research on the long-term impact of CSA and STE on mental health.

Estimates of the global prevalence of childhood sexual abuse (CSA) vary as a function of study design and informant source. While a large meta-analysis of predominantly retrospective self-report studies estimated a global lifetime prevalence of 11.8% (Stoltenborgh et al., 2011), a more recent population-based study relying on children’s self-reports show prevalence estimates of approximately 9% for contact sexual violence and 6% for completed forced sexual intercourse (Piolanti et al., 2025). Markedly higher prevalence rates has been observed in clinical samples (32%) (Pan et al., 2021). CSA victims may suffer from poorer quality of life in the life course since CSA is associated with a broad range of mental health problems (MHPs) in adolescence and adulthood, including anxiety, depression, and substance abuse. The relationship between CSA and MHPs has been documented in both cross-sectional (Kendler et al., 2000; Nelson et al., 2002) longitudinal, and meta-studies (Fergusson et al., 2008; Lindert et al., 2014). Furthermore, research has shown that CSA is strongly associated with later sexual dysfunction and sexual trade and exchange (STE) (Bagley & Young, 1987; Kaestle, 2012; Lavoie et al., 2010; Priebe & Svedin, 2009). Because studies have found that STE is associated with both CSA (Kaestle, 2012; Lavoie et al., 2010; Priebe & Svedin, 2009; Svensson et al., 2013) and later MHPs (Averdijk et al., 2019; Edwards et al., 2006; Krisch et al., 2019; Svensson et al., 2013), STE may play an important role in the association between CSA and subsequent mental health outcomes. STE may be viewed as a negative outcome associated with CSA and may also represent a form of sexual revictimization in a conceptual sense, in which repeated exposure to sexual exploitation contributes to an increased risk of MHPs. Experiencing repeated victimization, rather than a single episode, is associated with a greater risk of shame, blame, feelings of powerlessness, and maladaptive coping strategies (Classen et al., 2005). Building on this conceptual framework, the main aim of the present study is to examine whether STE serves as a mediating pathway between CSA and three common MHPs: anxiety, depression, and substance abuse. Given the low prevalence of CSA and STE in the general population, and the challenges of collecting prospective data on these groups, we rely on retrospective data from 335 Norwegian adolescents aged 12–20 recruited from the high-risk population of adolescents in residential youth care (RYC). Individuals are selected for RYC based on neglect, abuse, or conduct problems (Jozefiak et al., 2016; Kayed et al., 2015) and are expected to have higher rates of CSA, STE, and MHPs compared to the general population. The latter assumption has already been documented, as the sample was found to have a 76.2% prevalence of any DSM-IV mental health disorder, including a 34.0% rate of any anxiety disorder, 37.0% of any depressive disorder, and 11.9% of any substance abuse disorder (Jozefiak et al., 2016).

## Childhood Sexual Abuse, Anxiety, Depression and Substance abuse

Results of both single cohort and twin studies using diagnostic interviews for major depression and anxiety disorders report that CSA victims are at greater risk for developing anxiety and depression in young adulthood (Fergusson et al., 2008; Kendler et al., 2000; Nelson et al., 2002). Furthermore, the cohort study by Fergusson et al. (2008) and the twin-studies by Kendler et al. (2000); Nelson et al. (2002) also revealed that CSA predicted substance abuse in young adult victims of CSA. Two related explanations to why CSA victims are more likely to be pathologically anxious, depressed and use substances compared to their peers, is linked to the models of dissociation and experiential avoidance (EA). Dissociative reactions to trauma include the symptoms of dissociative amnesia, emotional numbing, and depersonalization, which serves as mechanisms to diminish awareness of distressing emotions (Friedman et al., 2021). Dissociation as a psychological defense mechanism creates distance from any distressing thoughts, feelings, memories or sensations related to the traumatic event. Dissociation is argued to be a form of EA – which is defined as a pathological process where an individual seeks to avoid, escape, or alter certain aspects of an experience (Hayes et al., 1996, p. 1154). In EA, actions are taken to alter the form, frequency, and context of the traumatic experiences, and is driven by the individuals need to disconnect from some specific private experiences such as bodily sensations, emotions, thoughts, memories, or behavioral predispositions (Hayes et al., 1996, p. 1154). Dissociation and EA may be central to explaining the relationship between CSA and MHPs among adolescents and young adults. As shown in a cross-sectional study of adolescents in residential treatment, dissociation fully accounted for the relationship between CSA and MHPs when included in the model, suggesting that dissociation may be an important mediator (Kisiel & Lyons, 2001). However, the role of STE in the developmental trajectories linking CSA to psychopathologies and substance abuse remains less explored.

## Childhood Sexual Abuse and Sexual Trade and Exchange

CSA is commonly defined as sexual activity involving a child that the child does not fully comprehend, is unable to consent to, or that occurs within a relationship characterized by power imbalance or exploitation (U.S. Centers for Disease Control and Prevention, 2024; World health Organization, 2006). In Norway, the legal age of sexual consent is 16 years, as stipulated in the Norwegian Penal Code (2005, § 196). CSA can include oral, genital, or rectal abuse, as well as exposure or viewing of sexual anatomy or pornography (Johnson, 2004). Numerous studies have found that CSA is related to an increased chance for the victims to engage in STE as they grow older (Bagley & Young, 1987; Bjørndahl, 2017; Garcia et al., 2024; Kaestle, 2012; Laird et al., 2020; Lavoie et al., 2010; Svedin & Priebe, 2007; Svensson et al., 2013). The Traumagenic Dynamics Model (TDM) by Finkelhor and Browne (1985) describes four typical traumagenic dynamics that may increase the risk for STE, and other negative mental health outcomes following CSA. These dynamics are (1) *Traumatic sexualization* following abuse, which shapes the child’s developmentally inappropriate and dysfunctional sexual feelings, (2) *Betrayal*, which results from experiencing harm inflicted by a trusted person who did not protect or believe in them, (3) *Powerlessness*, which arises from contraventions of the child’s will, desires, and boundaries, (4) *Stigmatization* is driven by inflicted negative responsibility, shame, guilt, and humiliation caused by abusers or others. All four dynamics contribute to a low self-perception, increasing the victim’s vulnerability to further abuse and dysfunction (Finkelhor & Browne, 1985). Findings from several studies support the TDM and show that individuals who have experienced CSA are more likely to experience stigmatization (Coffey et al., 1996). They may also experience difficulties in their relationships, lack trust in others, have low self-esteem, struggle with identity, and have problems with sexual functioning (Denov, 2004). Furthermore, CSA victims are also more likely to engage in high-risk sexual behavior, such as offering sexual services in exchange for compensation, including money, drugs, alcohol, clothing, or other goods and services (Krisch et al., 2019). One study found that youth involved in STE felt ruined after repeatedly disregarding their own boundaries, leading to carelessness and further exploitation (Bjørndahl, 2017). Powerlessness and lack of agency appear to be common among both victims of CSA and STE. Indeed, CSA appears to heighten vulnerability to STE, a finding supported by both in-depth qualitative studies and large representative population surveys (Bagley & Young, 1987; Bjørndahl, 2017; Kaestle, 2012; Lavoie et al., 2010; Svedin & Priebe, 2007; Svensson et al., 2013). Cross-sectional studies have shown that victims of CSA are three times more likely to engage in STE (Lavoie et al., 2010), and a study of youth involved in STE found that 63,8% of them reported a history of CSA (Fredlund et al., 2018). Finally, one study also revealed that 16.8% of adolescent girls who engaged in STE had at least once been forced to have sex (Edwards et al., 2006).

The above-mentioned associations between CSA and STE reported from cross-sectional and retrospective studies has also been confirmed in a large prospective cohort study matching 908 maltreated and 667 non-abused individuals over a time-range of 30 years (Wilson & Widom, 2008). Results revealed that those who experienced sexual abuse, physical abuse, and neglect in their younger years were more than twice as likely to engage in prostitution as they aged (Wilson & Widom, 2008). Given the consistent findings supporting a strong association between CSA and STE, we expect this pattern of associations to be reported in the present study based on data from a high-risk population of adolescents in RYC.

## Sexual Trade and Exchange and Mental Health Problems

The prevalence of STE among adolescents in the general population ranges from 0.5% to 5.0% across different populations and time periods (Edwards et al., 2006; Hegna & Pedersen, 2002; Kaestle, 2012; Lavoie et al., 2010; Martin et al., 2021; McNeal & Walker, 2016; Priebe & Svedin, 2009; Svedin & Priebe, 2004; Svedin et al., 2015). Several studies conducted in high risk populations have identified substantially higher prevalence estimates compared to general population studies; 8% (SiS, 2021), 15.8% to 37.7% (Head et al., 2021), and 8.1% (Decker et al., 2012). Furthermore, Gerassi et al. (2021) reported that 15.9% of the STE group had been in foster care, compared to only 1.9% of the total sample, indicating a substantially elevated likelihood of STE among youth with child welfare involvement.

Like the damaging traumatic experiences of CSA, STE is associated with a range of risk factors such as mental health problems (Bjørndahl, 2017; Svedin & Priebe, 2007), drug and alcohol abuse (Bjørndahl, 2017; Hegna & Pedersen, 2002; McNeal & Walker, 2016; Svedin & Priebe, 2007), and school absenteeism (Bjørndahl, 2017; Hegna & Pedersen, 2002) – all of which may have long-term consequences for future work prospects and income potentials.

Results of several cross-sectional studies suggest a relationship between substance abuse and increased likelihood of suffering from MHPs and STE among adolescents and younger adults. The results of one study of *n* = 4,339 individuals aged 16–23 revealed that those who engaged in sex work also reported significantly more symptoms of anxiety and depression (Svedin & Priebe, 2007). A second study based on *n* = 5,873 high school students reported that students who had sold sex were significantly more likely to report symptoms of anxiety and depression, and to be involved in drug use and binge drinking (Fredlund et al., 2018). Furthermore, Bjørndahl (2017) conducted a study to identify the reasons why young people engage in sex work, based on statements from their support system. The informants reported that adolescents in STE showed increased symptoms of anxiety and depression, as well as drug use (Bjørndahl, 2017). Finally, a cross-sectional study based on data from *n* = 12,000 13–16 year olds in Norway found that young people who had experienced STE reported significantly higher alcohol and drug-related problems (Hegna & Pedersen, 2002). Based on the evidence reviewed above, STE is likely to be associated with adverse mental health outcomes. We thus expected STE to be more frequent in our sample compared to the general population because of a higher probability of CSA. Based on previous studies, we also expected a positive association between STE and common MHPs.

## Mental Health Problems Specified

At the core of depressive disorders are dysphoric emotions and thoughts leading to helplessness and hopelessness, with withdrawal and isolation. At the core of anxiety disorders are fearful expectations and reactions that motivate protective or avoidant behaviors that interfere with daily functioning and quality of life (Ollendick et al., 2013). While depression is more oriented toward past loss and misfortune, anxiety focuses more on present or future risks. Anxiety and depression often co-occur in 20–40% of patients (Eysenck & Fajkowska, 2018).

Substance disorders are characterized by excessive use of psychoactive substances and use for emotional self-medication, leading to functional decline and substance dependence (Rutten et al., 2021). Substance abuse and intoxication can also be seen as experiential avoidance by numbing and sedating the person. Both anxiety and depression can motivate substance abuse or other maladaptive coping strategies such as experiential avoidance or partial mental dissociation, with increasing dysfunctional consequences (Loewenstein, 2018). Despite their relatedness, these mental health problems may be differently associated with trauma in general and CSA and STE in particular.

## The Present Study

The main aim of the present study is to explore differences between MHPs in the developmental trajectories involving CSA and STE. Based on the empirical evidence presented above, CSA victims seem to be more likely than the general population to engage in STE and to suffer from MHPs. However, it is still unclear from the current body of primarily correlational studies whether CSA alone trigger or increase substance abuse and mental health problems in later years, or whether the negative outcomes in adolescence and adulthood are mediated by STE as a form of revictimization. Therefore, we will examine how retrospective reports of CSA are related to intervening self-reported STE and current mental health problems, i.e., whether STE serves as a mediating pathway between CSA and future mental health problems, including substance abuse, among adolescents in residential care. These connections may be different for the three selected MHPs. Based on the existing literature, we consider the following research questions: (1) is CSA associated with STE, (2) is CSA directly associated with anxiety, depression, and substance abuse, and (3) is CSA indirectly associated with anxiety, depression, and substance abuse through the mediating variable of STE, and (4) is (2) and (3) different for the three MHPs.

## Method

### Participants

**Fig. 1:**
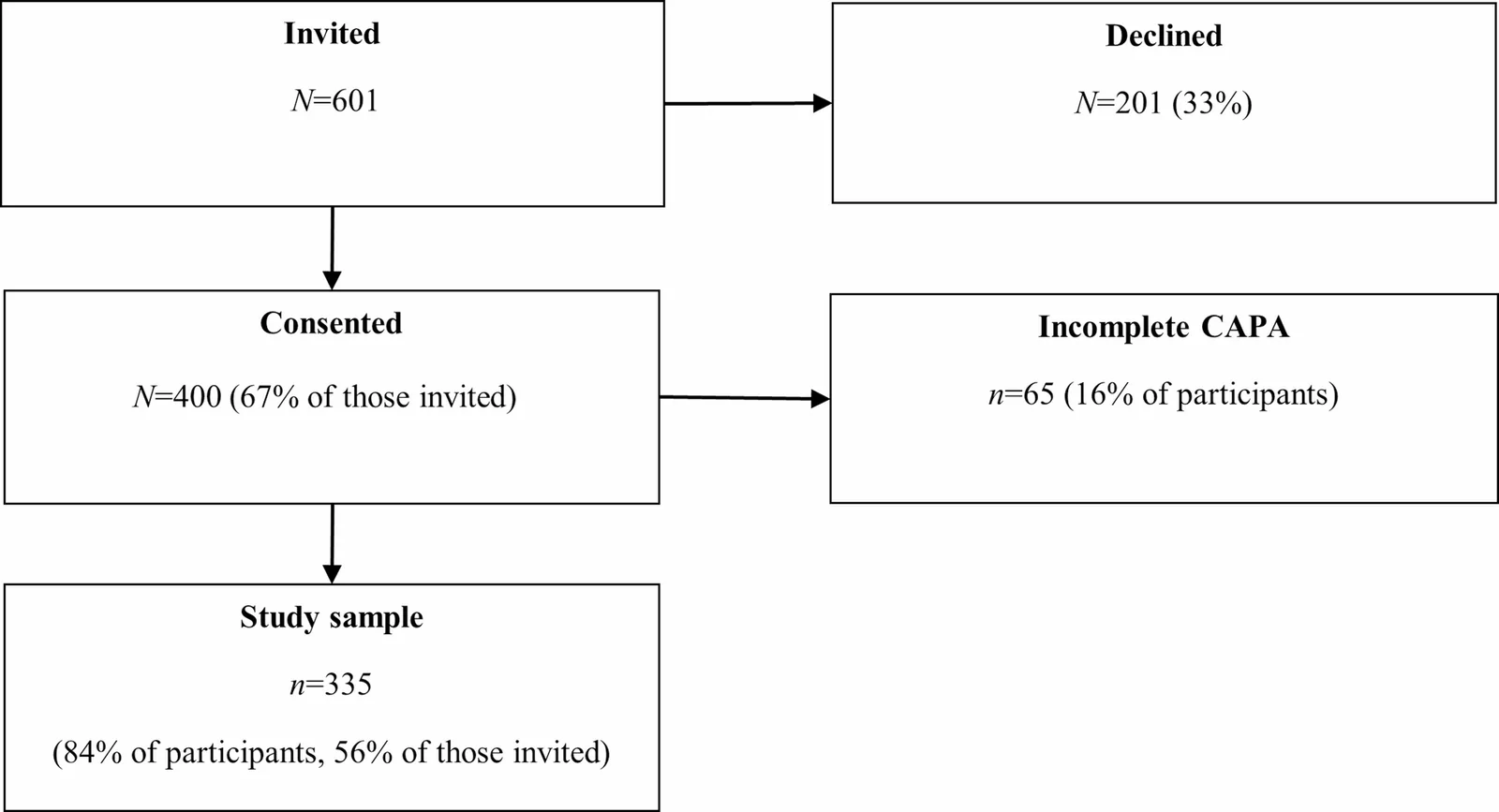
Flowchart of recruitment and exclusion citeria

All adolescents aged 12–23 years in residential youth care (RYC) in Norway were selected as the target group for a nationwide study on mental health and child protection history. There is a total of 98 RYC institutions in Norway, with *n* = 731 adolescents (potential participants). Prior to invitation to participate in the study, a total of 130 potential participants were excluded at an institutional or individual level. Those not invited to participate were youth in acute care and unaccompanied minors without asylum due to the expected high level of crisis. In addition, young people without Norwegian language skills and youth enrolled in MultifunC institutions (i.e. children and youth with the most severe problems on all or several domains of their lives such as home and family, school, and peer relationships) were excluded due to the high research burden. Finally, 601 young people in 86 RYC institutions were invited to participate (see Fig. 1). In 201 cases, parents or adolescents did not consent to participate, resulting in a total sample of 400 adolescents who participated in the data collection: a response rate of 67%. Of the 400 adolescents, 65 participants did not complete the Child and Adolescent Psychiatric Assessment (CAPA) and were therefore not included in the current study. Thus, our study includes 335 youth. For further information regarding data collection, attrition and imputation of missing data compared to observations, see Jozefiak et al. (2016).

 Table1 shows the demographic characteristics of subsample (*n* = 335), which the current study was based on. Compared to the total sample (*N* = 400), there were no substantial differences with regards to age, gender, placement and school status. The age of the participants ranged from 12 to 20 years (*M* = 16.35, *SD* = 1.33). Age at first placement ranged from 0 to 17 years (*M* = 12.55, *SD* = 3.90), and the number of placements ranged from 1 to 25 (*M* = 3.38, *SD* = 2.50).

**Table 1:** Demographic characteristics of the subsample

	subsample	
	*n*	%
Gender		
Boys	139	41.5
Girls	196	58.5
Are you in school today?		
Yes	226	67.7
No	108	32.3

*n* = 335

### Procedure

Data collection at the RYC sites was conducted by four trained research assistants who visited the sites where interviews and tests were conducted. Data collection at each facility was carried out over one to two days, depending on the number of local participants. The data collection lasted approximately four hours per participant, including the CAPA and additional interviews, as well as several questionnaires and standardized tests not included in the present study. Participants were allowed to stop or pause the data collection or withdraw from the research at any time, without providing a reason. Throughout the data-collection period, a team of child and adolescent psychiatrists and psychologists was available for emergencies. Each adolescent received a gift certificate of 500 Norwegian kroner (approximately USD 90), regardless of whether participation was discontinued. Data was collected between June 2011 and July 2014.

## Measures

### The Child and Adolescent Psychiatric Assessment (CAPA)

Information on the DSM-IV diagnostic categories, experience of sexual abuse (CSA), and sexual trade and exchange (STE) included in this study was collected using the CAPA (Angold & Costello, 2000). The CAPA is a semi-structured psychiatric interview that collects data on functional impairment, and the onset, duration, frequency, and intensity of symptoms of a wide range of psychiatric diagnoses that are combined into the cumulative categories used in this study: Any Anxiety Disorder, Any Depressive Disorder, and Any Substance Disorder. Interviewers were trained to conduct the categorical assessment based on in-depth probing following standardized baseline questions. Furthermore, they are trained to probe until it is possible to decide with certainty whether the symptom is present. Inter-rater reliability was assessed and documented (see Jozefiak et al., 2016 for details).

Experience of CSA were elicited through in-depth probing, beginning with the question “Has anyone ever touched you in improper places”? and for STE starting with “Have you ever had sex with someone to get something you wanted”? When sufficient information was revealed, the interviewer coded dichotomous variables for use in statistical analyses. These questions were also repeated, focusing on the last three months. This method provides research diagnoses with high validity, and moderate clinical validity. Other information used in this substudy, including school, healthcare and care history, was collected in a short additional interview designed specifically for the main research project (Kayed et al., 2015). This additional interview was conducted immediately following the CAPA interview which initiated the data-collection.

### Statistics

Crosstabulation was employed to explore the distribution of subgroups based on combinations of CSA, STE and MHPs. Specifically, it was used to identify how these combinations varied by current STE status, and to examine the overlap between CSA and STE categories. Pearson’s r was used to test significant correlations between the variables. Multivariate regressions within a structural equation framework were used to assess the relationship between CSA, STE, and MHPs. MHPs was regressed on CSA and STE, and STE was regressed on CSA to estimate the direct effects between the variables. In the same model, tests of indirect relationships from CSA, through the mediating variable of STE, and to each of the three MHPs of anxiety, depression and substance abuse were added. Because CSA, STE, MHPs are right-skewed dichotomous variables, a robust maximum likelihood estimator was used, which does not require multivariate normality and handles moderate deviations from normality well (Benson & Fleishman, 1994). A full information maximum likelihood procedure was used to handle missing data. Analyses were performed in Mplus 7.3 (Muthén & Muthén, 1998–2015).

## Results

### Associations Between CSA, STE and Mental Health Problems

Descriptive statistics for the study variables are presented in Table 2. More girls than boys reported MHPs, and especially higher rates of CSA, and STE. Only three boys reported STE. The exception to gender imbalance was substance disorder, where the same proportion − 12% of the boys and girls reported substance problems.

**Table 2:** Descriptive information on observed study variables by gender

	Total		Boys		Girls	
	*n*	%	*n*	%	*n*	%
Any Anxiety disorder						
Yes	117	34.9	38	27.3	79	40.3
No	218	65.1	101	72.7	117	59.7
Missing n=	0		0		0	
Any Depressive disorder						
Yes	125	37.3	31	22.3	94	48
No	210	62.7	108	77.7	102	
Missing n=	0		0		0	52
Any substance disorder						
Yes	42	12.5	17	12.2	25	12.8
No	293	87.5	122	87.8	171	87.2
Missing n=	0		0		0	
Sexual abuse						
Yes	89	27.5	9	6.8	80	41.9
No	235	72.5	124	93.2	111	58.1
Missing n=	11		6		5	
Sexual trade and exchange						
Yes	26	8.9	3	2.5	23	13.3
No	266	91.1	116	97.5	150	86.7
Missing n=	43		20		23	

*n* = 335

The prevalence of STE was notably higher among participants with a history of CSA (20.0%) compared to those without CSA (4.5%), as shown in Table 3. When examining mental health outcomes (Table 4), the presence of STE—regardless of CSA—was associated with a markedly increased prevalence of substance use disorders (53.8% across STE subgroups). In contrast, CSA alone was not associated with elevated rates of substance-related problems (5.8%).

**Table 3:** Crosstabulation between childhood sexual abuse (CSA) and sexual trade and exchange (STE)

	Missing STE		No STE		STE present		Total	
Missing CSA	2	40	2	40	1	20	5	100
No CSA	35	13.2	218	82.3	12	4.5	265	100
CSA present	6	9.2	46	70.8	13	20.0	65	100
Total	43	12.8	266	79.4	26	7.8	335	100

Diagnostic interview completely missing *n*= 65

**Table 4:** Crosstabulation of subgroups of CSA/STE combinations by present MHPs

	Any Anxiety Disorder				Any Depressive Disorder				Any Substance Disorder			
	No		Present		No		Present		No		Present	
	*n*	%	*n*	%	*n*	%	*n*	%	*n*	%	*n*	%
Missing	1	50	1	50	0	0	2	100	4	100	0	0
No CSA/STE	181	71	74	29	175	68.6	80	31.4	228	90.1	25	9.9
CSA only	28	53.8	24	46.2	26	50	26	50	49	94.2	3	5.8
STE only	6	46.2	7	53.8	6	46.2	7	53.8	5	38.5	8	61.5
CSA & STE	2	15.4	11	84.6	3	23.1	10	76.9	7	53.8	6	46.2
Total	218	64.3	117	34.5	210	61.9	125	36.9	293	86.4	42	12.4

*CSA *childhood sexual abuse, *STE *sexual trade and exchange, *MHPs *mental health problems (any anxiety disorder, any depressive disorder, any substance disorder); Missing = missing information regarding CSA or STE; diagnostic interview completely missing *n*=65

All percentages are calculated within each subgroup for participant with available information

*n*=335

Significant positive correlations were found between anxiety and depressive disorders (*r* =.41, *p* <.001), as well as between sexual abuse and both anxiety (*r* =.25, *p* <.001) and depressive disorders (*r* =.29, *p* <.001). Sexual trade and exchange was significantly associated with all other variables, most strongly with substance disorders (*r* =.40, *p* <.001) and sexual abuse (*r* =.30, *p* <.001).

**Fig. 2:**
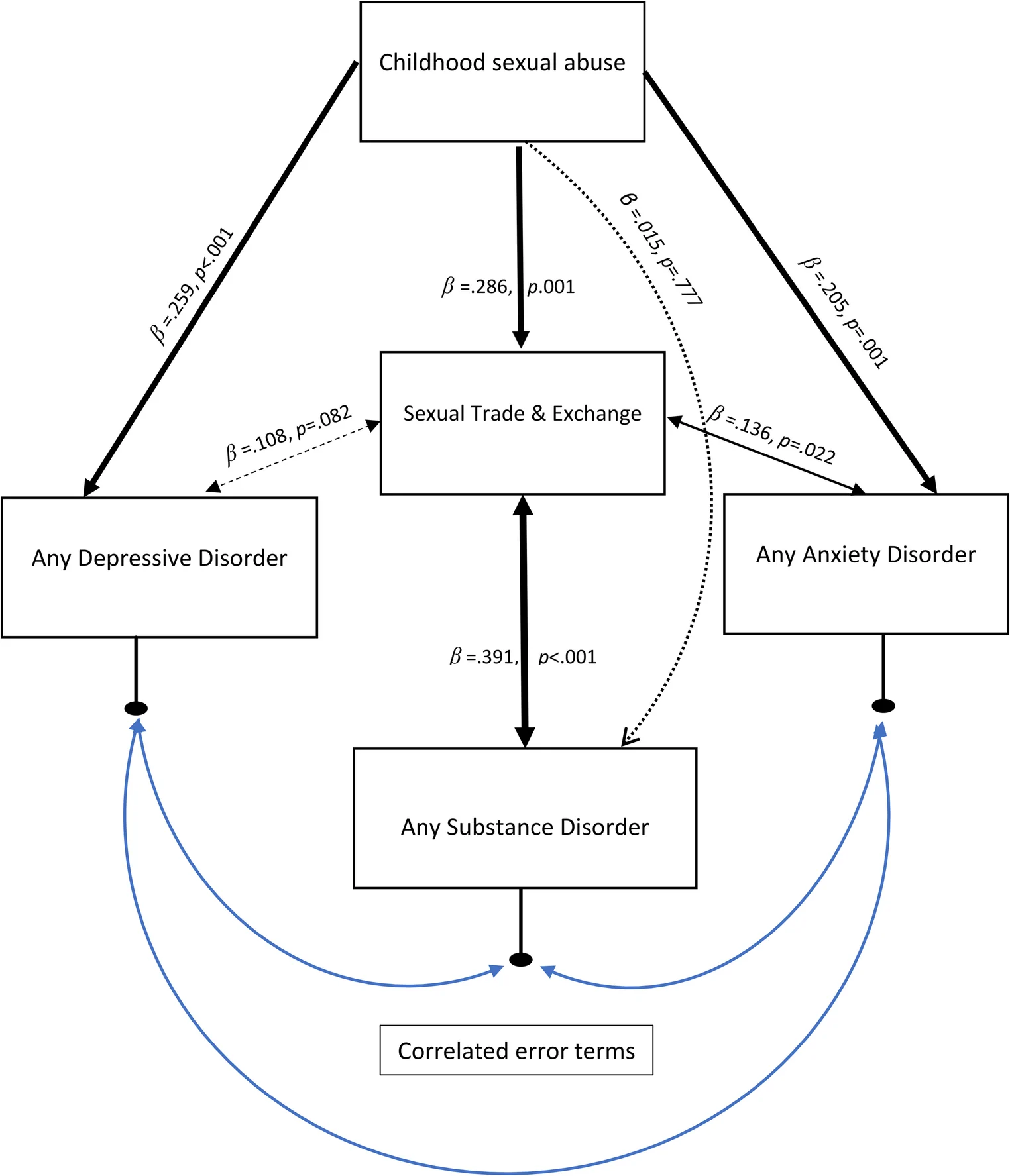
Path model of associations between CSA, STE, and MHPs.* Note*. *n*=335

Figure 2 shows the results of the multivariate regression analysis. CSA predicted anxiety and depressive disorders and future involvement in STE. CSA did not predict substance use disorders. Involvement in STE increased the likelihood that the adolescents would suffer from anxiety and substance disorders. The prediction of STE on depressive disorders was sub-significant with a *p*-value of 0.082.

### Indirect Associations between CSA and Mental Health Problems through STE, and Outcome-Specific Differences

The results of the indirect effects tested in the multivariate regression analysis are shown in Fig. 2; Table 5. The indirect effect of CSA via STE on any anxiety disorder was sub-significant, with a *p*-value of 0.059. The indirect effect of CSA via STE on substance disorders was significant. No significant indirect effect was observed for the outcome of depressive disorder. The estimated model was fully saturated, and the model therefore reached a perfect fit with the following fit indices: *χ*^*2*^ = 0.000, *df* = 0, *p*-value<0.001; RMSEA=0.000 (95% CI 0.000-0.000.000.000); SRMR=0.000; CFI = 1.000; TLI = 1.000. For each of the endogenous variables, a significant proportion of variance was explained by the model: Any Depressive Disorder, 9.5% of the variance (*R*^*2*^=0.095, *p*-value=0.005), Any Anxiety disorder, 7.6% of the variance (*R*^*2*^=0.076, *p*-value=0.011), Any Substance Disorder, 15% of the variance (*R*^*2*^=0.150, *p*-value=0.023), and STE, 8.2% of the variance (*R*^*2*^=0.082, *p*-value=0.027).

**Table 5:** Indirect effects of CSA through STE on any depressive disorder, any anxiety disorder and any substance disorder

Indirect effects	*β*	95% Cl	*p* value
CSA → STE→ Any depressive disorder	.031	-.01-.07	.101
CSA →STE→ Any anxiety disorder	.039	-.00-.08	.059
CSA →STE→ Any substance disorder	.112	.04-.18	.002

*n*=335

## Discussion

The aims of the study presented here were threefold, relying on retrospective data from 335 Norwegian adolescents aged 12–20 recruited from RYC. Our first aim was to examine possible associations between CSA and STE, and our results revealed that experiencing CSA was associated with an increased likelihood of engaging in STE during adolescence. Second, we tested for direct associations between CSA and common MHPs and found direct associations with anxiety and depression, but not substance abuse. Finally, we examined the possible mediating role of STE in the relationship between CSA and common MHPs and found an indirect connection from CSA via STE to substance abuse. The direct associations between STE and MHPs were strongest for substance abuse, significant for anxiety and sub-significant for depression. Thus, the three MHPs showed complex differences in their direct and indirect associations with CSA and STE.

The first result is consistent with earlier reports on the significant associations between CSA and STE (Bagley & Young, 1987; Bjørndahl, 2017; Kaestle, 2012; Lavoie et al., 2010; Svedin & Priebe, 2007; Svensson et al., 2013). Our second finding revealed that CSA was associated with anxiety and depression disorders to similar degrees, which is also supported by earlier studies (Fergusson et al., 2008; Kendler et al., 2000; Nelson et al., 2002). A mechanism that may contribute to the association between CSA and anxiety and depression is the experience of experiential avoidance. This is a common coping mechanism among children and youth who have experienced trauma. It involves seeking to avoid, escape, or alter some aspects of the painful experience (Hayes et al., 1996, p. 1154). Dissociation is an attempt to deal with emotional pain, but the consequence is rather an increase in anxiety and depression (Kisiel & Lyons, 2001) and is therefore a maladaptive coping strategy. Experiencing disconnection from one’s body and mind can lead to feelings of helplessness and sadness contributing to depression in addition to the feelings of betrayal, loss, and worthlessness typical for CSA victims (Miller & Seligman, 1975; Schauer & Elbert, 2010). In our study, CSA did not show a direct link to substance abuse, which differs from what earlier research has found (Fergusson et al., 2008; Kendler et al., 2000; Nelson et al., 2002). One reason for this may be that we also included STE, which seemed to explain the connection between CSA and substance abuse. Our results revealed that the group that had experienced CSA but not STE had the lowest rates of substance-related problems. By including STE in our analysis, we were able to separate it’s impact from that of CSA, something that previous studies did not account for.

While CSA was equally associated with anxiety and depression disorders, engaging in STE increased the likelihood of anxiety disorder and substance use disorder, but not depressive disorder. This finding suggests that STE increases the likelihood of anxiety beyond the influence of CSA, and that STE is the only factor in our model with significant influence on substance abuse. In other words; engaging in STE increases excessive fear and worry for anticipated negative events, which is typical for anxiety disorders (Ollendick et al., 2013). Furthermore, STE tends to increase substance abuse, which also is maladaptive and a potentially self-harming coping strategy that may maintain and increase symptoms of anxiety over the life course (Amendola et al., 2022). One reason for this could be that although STE and risky sexual behavior can be a way to alleviate stress in the short term, these types of coping strategies may perpetuate feelings of anxiety in the long term (Weiss et al., 2015).

The findings on the impact of STE on anxiety and substance abuse also help explain its mediating role in the relationship between CSA and substance use disorder. Contrary to previous findings (Fergusson et al., 2008; Kendler et al., 2000; Nelson et al., 2002), the present study did not find a direct association between CSA and later substance abuse. Instead, STE fully mediated this relationship. This suggests that CSA may not be sufficient to predict substance use disorder unless it is followed by further victimization, such as STE. In our sample, characterized by chronic abuse and neglect, CSA appear to increase vulnerability to revictimization. It is the subsequent experience of STE that is more proximally linked to substance abuse. This pathway reflects a cumulative strain process, consistent with General Strain Theory, where repeated and unresolved trauma leads to maladaptive coping strategies such as substance abuse.

According to Classen et al. (2005), substance abuse may be both a predictor and outcome of revictimization following CSA. Supporting this, Walker et al. (2024) found that substance abuse was associated with revictimization, but not with childhood maltreatment. Similarly, Walsh et al. (2014) reported elevated substance abuse among revictimized adolescent girls compared to those who were victimized only once or not at all. These findings underscore the importance of considering the broader context of cumulative trauma when examining pathways from CSA to substance use. The self-medication hypothesis – in which drugs are abused in order to cope with distress (Khantzian, 1997) – may be more relevant for revictimized adolescents. Adolescents can be more vulnerable both because of the relatively short time since both original traumas and later re-traumatization, but also given their psychological immaturity and the significant physical, mental, and social changes in the adolescent period. However, other background factors may result in substance disorders, indicated by that 25 of 42 (59.5%) of those in our sample with a substance disorder did not report CSA or STE. The substance disorder prevalence in this subgroup was only 9.9%.

### Strengths and Limitations

This study has several strengths, such as the use of validated diagnostic interviews to collect data on our central study variables. Furthermore, data collection among adolescents in residential care may be challenging due to their unstable and difficult living situation. Despite this, the present study succeeded in collecting data with acceptable participation rates. Most studies of childhood maltreatment and revictimization among youth rely on data from the student population, therefore, it is especially important to obtain data from other populations with higher prevalence of low-frequent factors that we wish to study. Finally, this is the first study to address CSA, STE, and MHPs in the same model, allowing to disentangle the relationship between these highly correlated domains.

Some limitations should be noted. The study is cross-sectional with a retrospective design for CSA and STE. Consequently, the design challenges the internal validity because of possible recall bias, confirmation bias and establishment of the temporal sequence of events (Grimes & Schulz, 2002). However, regarding recall- and confirmation bias, a significant agreement has been reported between prospectively recorded adverse childhood experiences compared to retrospective recalls of same events in adulthood (Reuben et al., 2016). The temporal sequence of events for CSA, STE and MHPs in the current study is not unambiguous. However, only three participants reported CSA during the last three months, and STE is less likely to occur before reaching adolescent years. Furthermore, MHPs were explicitly evaluated for the last three months before the interview but could have been present earlier. Consequently, the sequence CSA-> STE is therefore likely for much of our sample, while the sequence between STE and MHPs can be different for some participants. An example is that for some, STE may serve to enable substance abuse rather than leading to it. The STE-MHPs influences may also be bi-directional or circular, as Classen et al. (2005) suggest. Therefore, the typical or multiple temporal trajectories between CSA, STE, and MHPs should be clarified in prospective and qualitative studies. In addition, examining differences between youth who experienced trauma more recently and those whose experiences are more remote may help illuminate how temporal proximity shapes these complex associations.

Finally, the sample is skewed on gender, as only 3 boys (1.8%) vs. 62 girls (27%) confirmed STE. Hence, the findings reported here are primarily generalizable to the female population of adolescents in residential youth care.

### Conclusion and Implications

A central finding of the present study, based on data from adolescents aged 12–20 in RYC, is that the association between CSA and substance abuse disorder is fully mediated by STE, and STE alone is a stronger predictor than the direct and mediated influence of CSA on substance abuse disorder. Revictimization, a stronger stigma, shame, self-blame and the possibility for a victim identity may explain this potent influence of STE (Bjørndahl, 2017; Walsh et al., 2014).

Thus, our findings confirm the widespread belief that prostitution and its potential precursor STE is largely the result of post-traumatic victimization and injury rather than an immoral deviation and that substance abuse often is a dysfunctional coping strategy against shame and anxiety for further misfortune.

In the present study, there was an increased risk among CSA victims to become involved in STE. However, most victims do not take that path, but for those who do, there is a marked rise in problems like substance abuse and anxiety. Therefore, it is likely that helping CSA victims to avoid further involvement in STE may have a strong protective effect. One approach may be to focus on building self-worth and identity based on qualities other than sexual values.

## Data Availability

The data that support the findings of this study are not publicly available. However, any questions regarding the data can be directed to Tormod Rimehaug at tormod.rimehaug@ntnu.no.
